# Codevelopment of a Shared Decision-Making Resource Platform for Women and Girls in 3 Low- and Middle-Income Countries (ENGAGEEs): Protocol for a Participatory Research Project

**DOI:** 10.2196/78618

**Published:** 2025-10-21

**Authors:** Amédé Gogovor, Roberta de Carvalho Corôa, Tatiana Mossus, Coumba Gueye, Eliane Chaves Vianna, Monica Vieira, Babacar Mbaye Diop, Thierry Belleguic, Yves Tremblay, Farida Eunice Louré, Sabrina Guay-Bélanger, France Légaré, ENGAGEEs Group

**Affiliations:** 1 Department of Family Medicine and Emergency Medicine Faculty of Medicine Université Laval Québec City, QC Canada; 2 Department of Public Health, Faculty of Medicine and Biomedical Sciences Université de Yaoundé Yaoundé Cameroon; 3 Service de Cancérologie Centre Hospitalier National Dalal Jamm Dakar Senegal; 4 National School of Public Health Sergio Arouca Fundação Oswaldo Cruz Rio de Janeiro Brazil; 5 Joaquim Venâncio Polytechnic School of Health Fundação Oswaldo Cruz Rio de Janeiro Brazil; 6 Philosophy Department Cheikh Anta Diop University Dakar Senegal; 7 Department of Literature, Theatre and Cinema Faculty of Letters and Humanities Université Laval Québec City, QC Canada; 8 Department of Obstetrics, Gynecology and Reproduction Faculty of Medicine Université Laval Québec City, QC Canada; 9 VITAM - Center for Sustainable Health Research, Centre Intégré Universitaire de Santé et de Services Sociaux de la Capitale-Nationale Québec City, QC Canada; 10 Vice Dean for Research and Innovation Faculty of Medicine Université Laval Québec City, QC Canada; 11 See Acknowledgments

**Keywords:** shared decision-making, health decision-making, women’s health, low- and middle-income countries, decision support, decision aid, global health

## Abstract

**Background:**

Women and girls around the world face significant barriers in participating in health care decisions, particularly in low- and middle-income countries. Despite growing interest in shared decision-making (SDM), little is known about its implementation in these countries, and no rigorous assessment of the decision-making needs of women and girls in these contexts has been conducted. There is little SDM training and there are few decision-support tools (DSTs) specifically designed for health care decision-making by women and girls in these countries. DSTs (paper or digital) present options and probabilities and help users articulate their values and preferences.

**Objective:**

We aim to codevelop an SDM resource platform, including locally relevant DSTs and instructional materials, to support SDM with women and girls in Brazil, Cameroon, and Senegal.

**Methods:**

We will conduct a 4-phase participatory research project. We will use the Gender-Based Analysis Plus tool to support inclusive participatory research throughout the study. First, we will follow the Integrated Knowledge Translation framework to form a local steering committee in each country, including patients and community representatives, health and social service professionals, and decision-makers. Second, we will follow the Ottawa Decision Support Framework to conduct individual interviews and focus groups to identify the decision-making needs of women and girls. We will include 20 women and girls, along with their family members, 15 health and social service professionals, and 15 representatives of community-based organizations in each country (n=150). The Double Diamond human-centered design framework will be used to codevelop a digital SDM resource platform where DSTs and instructional materials for SDM training can be made accessible. Finally, we will assess the scalability of the platform by using the Innovation Scalability Self-Administered Questionnaire. We will report our study by using the Standards for Reporting Qualitative Research guideline and the Guidance for Reporting Involvement of Patients and the Public.

**Results:**

Financial support for this project was received on February 1, 2023. This protocol was submitted during data collection but before analysis. Data collection began in January 2025. By July 2025, 92 of the 150 participants had been recruited. We expect to publish our results in December 2026.

**Conclusions:**

Ultimately, more women and girls from low- and middle-income countries will be involved in health care decisions, and more clinical teams will be able to integrate SDM into their care practices. The local steering committees will ensure equitable partnerships and the SDM resource platform will promote more inclusive approaches to SDM worldwide. The scalability assessment will help us plan to expand the impact of the platform to other regions.

**International Registered Report Identifier (IRRID):**

DERR1-10.2196/78618

## Introduction

Health care decisions have traditionally been unilaterally made by health care providers or clinical teams [1]. Shared decision-making (SDM) has proved to be an effective practice for changing this power dynamic [2]. SDM is a collaborative process in which clinical teams and patients engage together in choices related to health decisions, considering the best available evidence and the values and preferences of the person receiving care [3]. It has a beneficial effect on reducing health inequalities, as underserved populations have been shown to benefit the most [4]. Decision-support tools (DSTs) (eg, decision aids, personal decision guides, conversations aids) and the training of health professionals can facilitate SDM [5,6].

DSTs enable patients and professionals to adopt the behaviors essential to SDM. They can be used before, during, or after a consultation, and their use has proven effective in reducing decisional regret [5,7]. However, DSTs are mostly implemented in high-income countries, particularly in the United States, Canada, and Europe, and little is known about how they could support SDM in low- and middle-income countries (LMICs) [5]. Moreover, existing DSTs and instructional materials for SDM training are not tailored to LMICs and are rarely developed with health care stakeholders from these countries.

**Figure 1:**
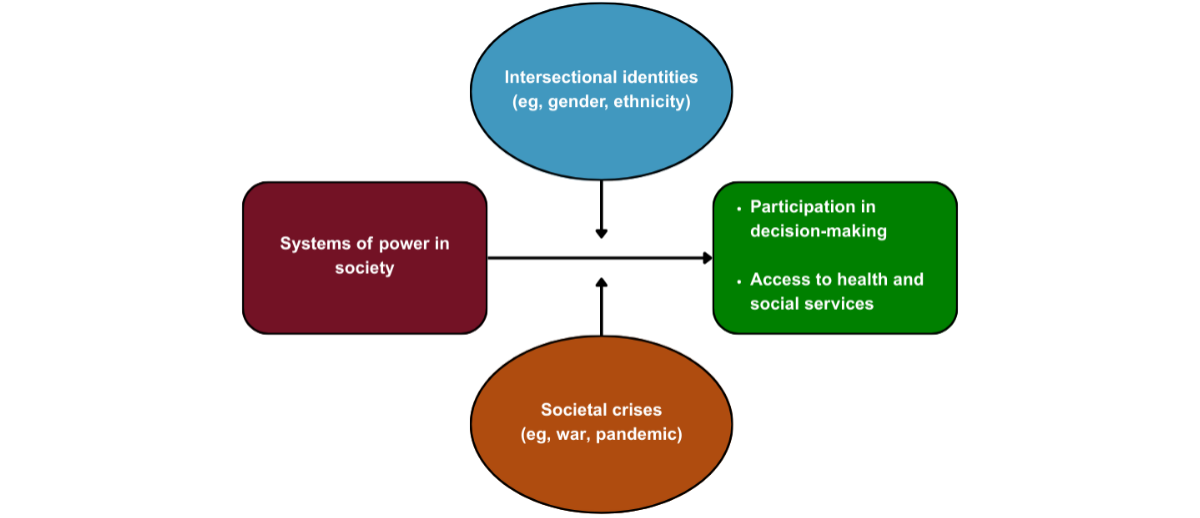
Power dynamics in society and participation in decision-making.

The research priorities set out in the United Nations Research Roadmap for the COVID-19 Recovery include helping eliminate discrimination (eg, racism, gender, poverty-based exclusion) in service delivery by engaging diverse stakeholders so that health systems become drivers of equity in society [8]. The document emphasizes the importance of engaging women and girls from LMICs in health care, since they are disproportionately excluded from decisions about their own health [9]. For example, a recent survey on maternal health decision-making in Nigeria showed that health care decisions were made by the husbands of 90% of the participants [10]. Women's and girls' unequal access to and engagement in health care services is based in part on unequal power relations shaped by sex and gender norms, including the division of labor between men and women (Figure 1) [11]. There is therefore an urgent need to develop interdisciplinary research for scaling SDM interventions so that more women and girls can benefit from them, especially in the context of LMICs.

As gender power dynamics and societal expectations put women and girls at the greatest risk of vulnerability, consideration of sex and gender is recommended as an entry point to a better understanding of the complex and converging factors that influence access to health services and affect involvement in health care decisions among systematically underserved groups [11]. For example, gender norms embedded in families and cultures can affect women’s involvement in decisions about their health, as well as the advice given, options offered, and quality of care provided [12]. However, to fully capture the complexity of the experience of women and girls, an intersectional analysis is essential—one that accounts for the interrelated social determinants of their involvement in decision-making, such as age, geography, ethnicity, socioeconomic status, marital status, and cultural beliefs [13]. Taking a human rights perspective acknowledges that all these intersecting factors may contribute to impeding the ability of women and girls to freely make informed decisions about their own health, essential for their human dignity as well as for their health and well-being [14]. Therefore, there is a need to create initiatives and partnerships that can enable this group to make informed decisions about their health and well-being.

In general, the development of DSTs and SDM training programs is guided by the decision-making needs of a targeted group [15]. Decision-making needs may be defined as factors that prevent people from making informed health care decisions. They are usually identified in relation to a specific decision point (eg, a choice faced in the context of a specific health condition, treatment, screening) and may include inadequate knowledge, uncertainty, unclear values, or a lack of support and resources to make an informed decision [15]. To the best of our knowledge, no systematic assessment of the decision-making needs of women and girls in LMICs has been undertaken.

**Figure 2:**
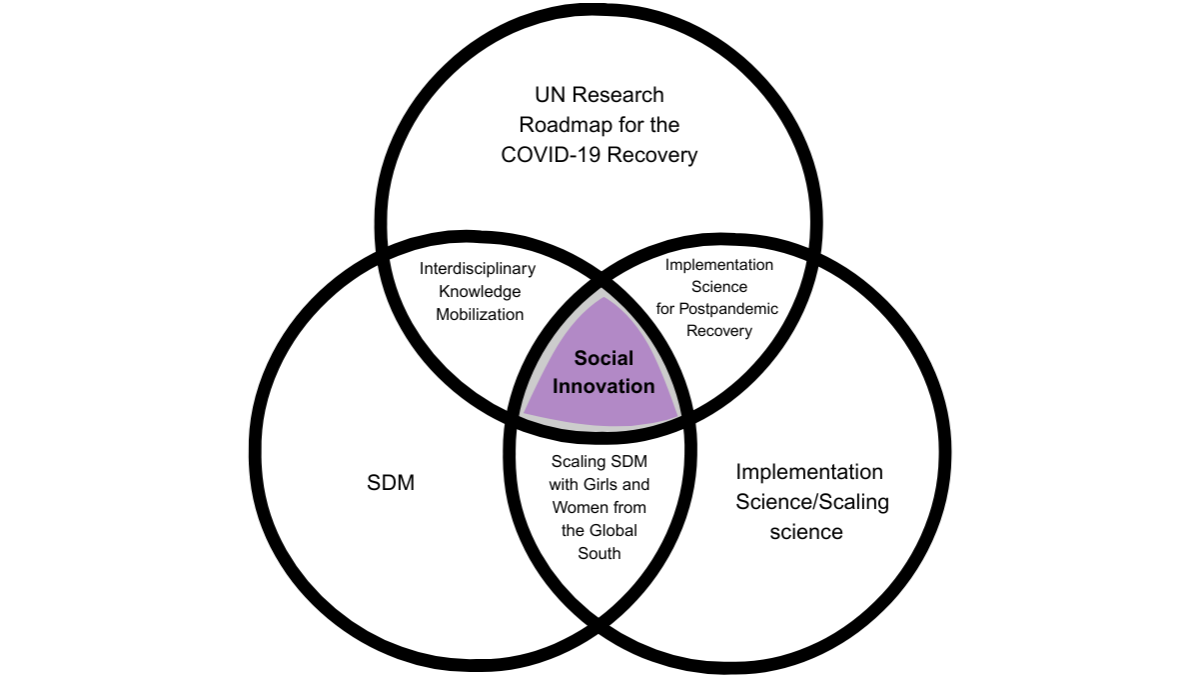
Social innovation based on key areas of the United Nations (UN) Research Roadmap for the COVID-19 Recovery blueprint. SDM: shared decision-making.

In line with the priorities of the United Nations Research Roadmap for the COVID-19 Recovery (Figure 2) and in collaboration with partners in Francophone Africa and Latin America, the ENGAGEEs (*ENGAger les femmes et les filles du Sud Global dans les décisions concernant leur santé et leur bien-être pour la mise en œuvre de la décision partaGÉE*, or Involving women and girls in decisions about their health and well-being through SDM) project aims to codevelop a digital resource platform for supporting SDM among women and girls in Brazil, Cameroon, and Senegal. The specific objectives of the project are to (1) establish an equitable and sustainable partnership with partners in the 3 countries, (2) identify decision-making needs in terms of the health and well-being of women and girls in these countries, (3) codevelop an SDM resource platform that meets these needs, (4) explore strategies for scaling SDM in the 3 countries through a scalability assessment of the codeveloped SDM resource platform, and (5) build SDM capacity in the 3 countries.

## Methods

### Context of the Study

The project coordination committee is based in the province of Quebec in Canada. It is led by 2 early-career researchers from Togo and Brazil, with mentorship from a Canadian senior researcher with 20 years of expertise in SDM. The research team based in Canada also includes one research professional and one student, both from Burkina Faso. The decision to perform the project in 2 African countries (Cameroon and Senegal) and 1 Latin American country (Brazil) was based on the knowledge, experience, and networks of the team members, who identified local research teams interested in SDM and who had access to the resources necessary for the development of the study. It is worth noting that Rwanda was originally included in the project. However, it was withdrawn because the researcher associated with the grant application and designated as the local project leader had been promoted to a position within the Rwandan government and was no longer available to continue implementing the project. The research team informed the funding agency, which approved the withdrawal.

### Overall Methodology

The ENGAGEEs project will take place in 4 phases: (1) forming local steering committees to establish equitable and sustainable partnerships in the 3 countries, (2) conducting individual interviews and focus groups to identify the decision-making needs related to the health and well-being of women and girls in the 3 countries, (3) codeveloping the SDM resource platform by using a human-centered design approach, and (4) assessing the scalability of the codeveloped platform by using an evidence-based scalability assessment tool. Each phase corresponds to a specific objective and methodology. Building SDM capacity is a cross-cutting objective throughout the project.

### Gender-Based Analysis Plus

Our team is experienced in integrating sex and gender considerations into implementation research on SDM [16,17]. We will apply the Gender-Based Analysis Plus (GBA Plus) tool at all phases of the project [18]. To mitigate the risk of reproducing inequalities in the project and to ensure the inclusion of groups that are systematically disadvantaged, such as women and girls in LMICs, we will mobilize additional efforts and targeted strategies. These include, for example, engaging community representatives to support recruitment, using plain language in communications and questionnaires, assembling interdisciplinary teams, and allocating extra resources to respond to situations involving gender-based violence. Thus, when designing project activities, we will apply the GBA Plus tool to the composition of the governance structures and the selection of participants, helping us to (1) reflect on and make informed methodological and analytical decisions in light of the expected impacts of the project on women and girls, as well as on health care stakeholders in the 3 countries; (2) identify which activities should be tailored to meet their diverse needs; and (3) discover, anticipate, and mitigate any barriers preventing women and girls from accessing, participating in, or benefiting from the project. In data collection and analysis, in each country, we will operationalize GBA Plus and the intersectional approach through the collection and disaggregation of data according to the PROGRESS Plus variables: gender, place of residence, race, ethnicity, language, occupation, religion, education, socioeconomic status, and social capital [13,19,20]. We will use the Sex and Gender Equity in Research guidelines to ensure appropriate reporting of gender-related information in study design, data analysis, results, and interpretation of findings [21].

### Phase 1: Partnership Development and Establishment of Steering Committees

#### Study Design

**Figure 3:**
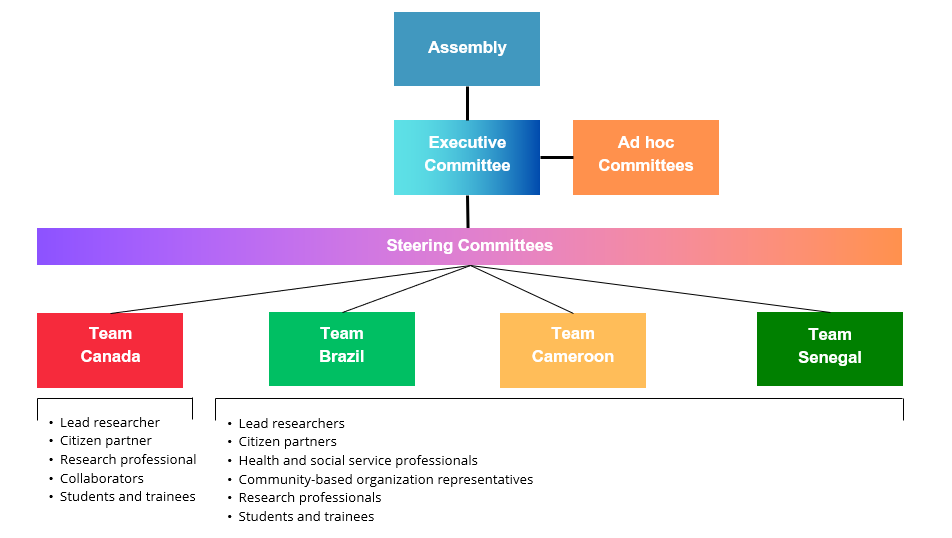
Project management structure.

We took 5 months to implement this phase. We followed the Integrated Knowledge Translation model to design the project’s governance structures [22]. The model recommends that knowledge users be involved in project development as equal partners from the beginning, ensuring the relevance and the usability of the knowledge products of the research. Our Integrated Knowledge Translation strategies included (1) an assembly made up of members of the international project team, including collaborators who participated in securing the funding; (2) an executive committee formed of the heads of each country team; and (3) local steering committees and local research teams in each country (Canada, Brazil, Cameroon, and Senegal). The local steering committees ensure that health care stakeholders, including women and girls who will be impacted by the project, are involved at all stages of the ENGAGEEs research project. The project governance is presented in Figure 3.

#### Participants

The assembly is composed of researchers, citizen partners, and collaborators (eg, policy decision-makers and health and social professionals) who participated in designing the project and securing the funding. It is an interdisciplinary team (eg, philosophy, medicine, psychology) with diverse expertise (eg, social justice, women’s health, scaling of innovations) (See ENGAGEEs group in the Acknowledgments section). The executive committee is composed of the researchers leading the project in each country and the research officers supporting the development of project activities.

For the local steering committees, we included health care stakeholders involved in the project and in the health and well-being of women and girls, including themselves and their family members. We targeted health and social service professionals, community representatives, researchers, and decision-makers, ensuring that at least one person from each of these groups was present on the local steering committees. Local research teams were also formed of project researchers, research officers, and students. For interviewing, we only engage local partners who are trained in qualitative research techniques such as how to ensure participants’ psychological safety during interviews and how to mitigate interview bias. If they are unqualified in these techniques, we offer training. We used the Guidance for Reporting Involvement of Patients and the Public guideline to report on the involvement of these stakeholders in the project.

#### Ensuring an Equitable Partnership

The project’s governance is structured around strategies to facilitate shared learning and action. To ensure an equitable partnership, we adopted the following strategies.

First, each research team was provided with the financial resources necessary for the development of project activities and is responsible for managing them, making their own decisions according to their local context. This strategy aimed to ensure that research teams have a sense of ownership over the project and autonomy in conducting the research with reference to the particularities of their own contexts. Second, each local research team built on existing networks and strategies in their own country to involve health care stakeholders and women and girls in the project. For example, local research teams relied on community health agents who are essential staff in primary health care in the countries of the project to identify and invite a woman or girl to take part in the steering committee. Third, we conduct monthly meetings of the executive committee, including the local research teams, to share decisions on the project’s direction, follow up on activities, and present preliminary data.

We use communication platforms that are most suitable in the local contexts (eg, mobile phones, social media) and will contribute to upgrading communication equipment in country teams where necessary. To accommodate different time zones, we adapt schedules to the various geographical areas. All project documents are provided in both French and Portuguese. We combine human assistance with online translation tools for translating documents and meetings. In rural areas where populations speak local languages, members of the local research team provide simultaneous interpretation if necessary. If not, they engage local interpreters who speak both the local and the official languages. During the meetings, dedicated time is set aside for each country’s team to speak, and every contribution is documented. In addition, the Canadian team will visit each country to engage with local stakeholders and discuss research needs in SDM. A visit to Canada by researchers from all countries is also planned to share the project’s initial results, challenges, and proposed solutions. The training modules offered by the Quebec Support for People and Patient-Oriented Research and Trials Unit will be presented to all project stakeholders, with the option of translation into other languages (eg, Portuguese) [23].

### Phase 2: Identification of the Decision-Making Needs of Women and Girls in Brazil, Cameroon, and Senegal

#### Design

Phase 2 is scheduled to last 6 months. To identify the decision-making needs of women and girls, we will conduct a qualitative descriptive study [24] guided by a pragmatic vision of the world and adopting a pluralist position consisting of collecting more than one type of data in order to best answer the research question [25]. In the SDM literature, decision-making needs are generally associated with a specific decision point (eg, about a health condition such as diabetes or about a screening procedure such as a breast cancer diagnostic test). However, in the context of the ENGAGEEs project, these needs may not be limited to a single decision point. Instead, they may reflect foundational needs required to support the practice of SDM, such as women’s and girls’ awareness of their right to participate in health care decisions or clinical teams’ knowledge of the steps and benefits of SDM. The Standards for Reporting Qualitative Research guideline will be used to report the qualitative research [26].

#### Study Population

We will include 20 women and girls along with their family members, 15 health and social service professionals, and 15 representatives of community-based organizations in each country (n=150) [25].

#### Eligibility Criteria

The eligibility criteria will be as follows.

1. Eligible women and girls will be 18 years of age or older, will have encountered difficulties in accessing health services or are interested in the subject, and will be available to participate in a 1-hour interview or a focus group session.
2. Eligible health and social service professionals are those willing to work in an interprofessional team.
3. Eligible representatives of community-based organizations are those willing to work in an organization that develops actions with women and girls aimed at improving their health and well-being.

#### Recruitment

Local teams will recruit participants in health and social service facilities (eg, family planning services, community organizations and associations working for the health and well-being of women, networks of associations working to promote women’s rights, hospitals). The local research teams and steering committees will use their networks to identify representatives of community organizations that include women and girls who may not have access to health and social services. Recruitment strategies will include placing posters advertising information sessions and actively recruiting through word of mouth, networking, and the snowball method. Local research teams will monitor recruitment using the PROGRESS Plus variables to ensure inclusivity and to verify that underserved groups are adequately represented in the participant samples [25].

#### Data Collection

Following the Ottawa Decision Support Framework, we will conduct semistructured individual interviews and focus groups to identify the decision-making needs of women and girls [27]. Based on existing literature in the field and principles of data saturation [28], we determined that a minimum of 50 participants per country—at least 20 women, girls, and their family members, 15 health and social service professionals, and 15 representatives of organizations—will be sufficient. The steering committee’s expertise will be mobilized to ensure a variety of participant profiles.

Prior to the interviews and focus groups, participants will watch a short video on SDM to allow them to better express their experiences in decision-making concerning their health. We will primarily use existing publicly available SDM videos, and local adaptations will be made by local teams as needed [29]. The viewing will be preceded by a brief introduction, followed by a time of sharing and discussion. Note-taking and/or recordings of these sessions will be performed by a research team member, and the analysis of this data will enrich project documentation and the development of SDM resources and tools for disseminating the project’s results. Interviews and focus groups will last approximately 1 hour and will be held either in person or online and recorded with the consent of the participants. The consent form must be completed before starting the data collection. Because of the vulnerability of this population and the sensitivity of some of the topics discussed, such as domestic violence, particular attention will be paid to managing the risks of interviewer bias, social desirability bias, and ensuring data reliability.

To minimize interviewer bias, interviewers will be trained to approach subjects with empathy, respect, and good humor, and to receive responses neutrally. To minimize social desirability bias, we will collect more than one source of information, triangulate the data, and identify socially desirable responses. The researchers will conduct interviews and focus groups only after gathering information from document analysis. We will include time to develop a relationship of trust with the interviewee to develop confidence and encourage unbiased answers. Data collection will take place in a comfortable and private location, and interviewees will be reassured about the anonymity and confidentiality of their personal information. However, in some political contexts, social desirability bias is inevitable due to social and political repression. In these cases, the researchers will not avoid it but will include it in their analysis and discussion [30].

We will incorporate standardized data collection tools, adapted as necessary for cultural appropriateness. Supervision and regular debriefing sessions, including steering committee meetings, will be conducted. Based on the elements of PROGRESS Plus, information on the sociodemographic and professional profile of the participants will be collected using a questionnaire at the beginning of each meeting. Interviews will be conducted in the official languages of each country, and if necessary, in participants’ mother tongues, by members of the project team in each country.

#### Data Analysis

Descriptive analyses will be conducted for sociodemographic data, which will be disaggregated according to the PROGRESS Plus variables. For qualitative data, interviews will be transcribed verbatim and, if necessary, translated into French. The anonymized transcripts will be uploaded to NVivo software (QSR International) for analysis. To categorize the decision-making needs and available services, information, and support opportunities, we will conduct a thematic analysis. We will perform a mixed analysis by using both an inductive and deductive approach. We will create themes based on the Ottawa Decision Support Framework (deductive approach) and explore the decision-making needs in relation with elements of intersectional and contextual factors to identify emerging themes (inductive approach) [27,31]. A preliminary list of the themes is provided in Table 1.

The decision-making needs identified will be classified based on their frequency in interviews and focus groups. Results will be presented in tables for each country separately as well as in aggregate form. To support the dissemination of findings to lay audiences, we will use concept maps and figures. The data will be written in plain language and summarized in a report format.

**Table 1:** Decision-making themes from the Ottawa Decision Support Framework [27].

Categories		Themes
**Needs assessment (determinants of the decision)**		
	Perception of the decision	Knowledge Decision-making conflict Decision-making steps Predisposition
	Perception of others	Roles in decision-making
	Resources needed for decision-making	Personal: previous experience, self-confidence, motivation, decision-making skills External: support (information, advice, moral, material, financial, professional aid) from social assistance networks and organizations
	Characteristics	Client: age, sex, marital status, education, occupation, culture, residence, medical diagnosis and prognosis, health status Practitioner: age, gender, education, specialization, culture, practice situation, experience, counselling style
**Provision of decision support**		
	Providing access to information	Health status Options
	Provision of advice and support	Communicating with others Access to support and resources

### Phase 3: Codevelopment of the ENGAGEEs SDM Platform

#### Design

We anticipate 9 months to implement this phase. The Double Diamond human-centered design framework will guide the codevelopment of the ENGAGEEs SDM platform [32,33]. We will use an iterative process that includes exploring the decision-making needs of women and girls (discover phase); identifying their decision-making priorities (define phase); co-designing DSTs, SDM instructional materials, and the layout of the ENGAGEEs SDM platform (codevelopment phase); and testing and evaluating the ENGAGEEs SDM platform with local stakeholders (deliver phase) [32,33]. The whole process will be conducted through online workshops and local working groups composed of members of the research teams, steering committees, and collaborators in each country.

#### Exploring the Decision-Making Needs of Women and Girls

The results of phase 2 will be discussed by the project stakeholders (assembly, committees, and research teams) in a 1-day online workshop. The aim of this workshop is to understand the decision-making needs of women and girls in the 3 study countries and to discuss common findings as well as contextual differences. First, as a preparatory activity, the report produced in phase 2, including tables, concept maps, and figures, will be sent to participants 2 weeks before the meeting. Second, during the workshop, each local research team will present the health care context in their country, the data collection context, project development activities, and the results of phase 2. Third, an open discussion session will be held to exchange impressions and insights. A report of this workshop will be prepared by the research team based in Canada and sent to all participants within 2 weeks.

#### Identifying Decision-Making Priorities

Upon receiving the report from the first workshop, local research teams and steering committees will be asked to meet and form working groups to classify and prioritize the decision-making needs of women and girls in their country. The results of the local working group discussions will then be presented in a second 1-day online workshop, during which project stakeholders will discuss the decision-making needs and priorities to be addressed by the ENGAGEEs SDM platform. Prioritization will be based not only on the importance of each decision-making need but also on the feasibility of codeveloping DSTs and instructional materials that address them for inclusion on the platform. These priorities may be specific to one country or reflect the common needs identified across all 3 countries. A report of this workshop will be prepared by the research team based in Canada and sent to all participants within 2 weeks as well.

#### Co-Designing DSTs

This step includes identifying the evidence-based data, writing the draft version of the DSTs, and evaluations of the draft by the local steering committees. At least one DST (eg, option grids, videos, decision aids) will be drafted for each country by local working groups with support from the team in Canada. First, the Canadian research team will search scientific databases and the Ottawa Decision Support Framework Decision Aids Library Inventory to identify potential DSTs that can be adapted and improved to address the decision-making needs prioritized by the ENGAGEEs project [34]. The local working groups may also contribute to this process by complementing the search and developing their own resources if none of the existing DSTs match their needs. Second, working groups will be requested to write a draft version of their proposed DSTs. Third, the Canadian team will meet with each local working group to review the strengths and weaknesses of proposed DSTs, consider relevant contextual factors, and determine appropriate formats for the resources. Finally, women and girls, along with health and social service professionals from the local steering committees, will evaluate the codeveloped DSTs and propose modifications if needed. The final versions of the DSTs will be provided in French, English, and Portuguese, and will be reviewed for comprehensibility by a literacy service.

#### Co-Designing Instructional Materials

This step will be led by the Canadian research team. The Canada Research Chair in Shared Decision Making and Knowledge Mobilization developed and keeps a directory of evidence-based SDM training courses [6,35]. Several of these courses for health and social service professionals have been evaluated [36]. In addition, the team itself has many years of experience with SDM training, including SDM workshops for the public. We will select instructional materials from existing training programs and enrich them with the contextual findings from phase 2. The aim is to inform both clinical teams and women and girls in Brazil, Cameroon, and Senegal about the essential elements and necessary skills for SDM. The instructional materials will include information on SDM and related tools presented through short texts, illustrations, and/or video links. The Canadian research team will use an iterative process to review and approve the instructional materials in collaboration with the local steering committees. The instructional materials will be made available in French, English, and Portuguese.

#### Co-Designing the ENGAGEEs SDM Platform

We will co-design the layout of the ENGAGEEs SDM platform through an ideation and cocreation process involving local research teams and local steering committees in collaboration with technical experts. The goal is to integrate the developed resources and ensure their usability for end users, including layouts for smartphones and tablets. The Canadian research team will work with digital development experts at Université Laval to present the goals of the ENGAGEEs SDM platform, define user profiles, and share the materials to be included (DSTs and instructional materials). Based on this input, the experts will develop the first prototype of the platform. This prototype will be presented to project stakeholders for feedback and suggestions for improvement. This process will continue until a satisfactory and usable version is achieved. The platform will be developed in French and Portuguese, the official languages of the participating countries, and will contain all the developed project resources. The translation of the project deliverables will be performed by bilingual researchers and professional translators on the project team to ensure the accuracy and quality of the content.

#### Testing and Evaluating the ENGAGEEs SDM Platform

We will adopt a mixed methods approach that combines validated quantitative instruments with participatory qualitative techniques to test and evaluate the ENGAGEEs SDM platform. We will use the System Usability Scale, a widely recognized 10-item questionnaire that provides a global measure of perceived usability for digital platforms [37]. In addition, the Health Information Technology Usability Evaluation Scale will be applied, as it is specifically designed to assess usability in health technology contexts, including perceived usefulness, ease of use, and user control [38]. The evaluation will involve women and girls as well as health and social service professionals who are members of the local steering committees. To complement the quantitative data, guided discussions will be conducted by the local research teams to gather feedback on platform content, accessibility, and contextual relevance. Notes will be taken during these sessions to capture participants’ reflections, perceived barriers, and suggestions for improvement. Both types of data will be triangulated to enhance comprehensiveness and to ensure that the platform is user-centered, contextually appropriate, and responsive to the needs of key stakeholders.

### Phase 4: Assessing the Scalability of the ENGAGEEs SDM Platform

Six months have been allocated for the implementation of this phase. We will use the Innovation Scalability Self-Administered Questionnaire (ISSaQ 4.0) to assess the scalability of the ENGAGEEs SDM platform, including its resources (DSTs and instructional materials). ISSaQ 4.0 is a scalability assessment tool that integrates considerations of sex, gender, equity, diversity, and inclusion. It consists of 37 items divided into 12 components deemed essential for scaling health and social innovations [39]. The questionnaire will be completed by the local steering committees who will reflect on the potential and strategies for scaling the ENGAGEEs SDM platform in their respective countries, with the goal of increasing its impact and supporting SDM among a broader and more diverse group of women and girls. We will assure long-term sustainability of the platform with the following strategies.

1. The initial version of the platform will be built using a free web-based collaborative tool, thereby minimizing initial capital expenditure and recurring licensing costs. This choice enhances affordability, flexibility, and ease of customization for future needs.
2. A sustainability plan will be developed to explore local resources for long-term ownership through integration into existing institutional systems.

### Ethical Considerations

Ethics approval was obtained from the ethical boards of each country: Comitê de Ética em Pesquisa da Escola Nacional de Saúde Pública and Comissão Nacional de Ética em Pesquisa of Brazil (76227523.6.0000.5240), Comité Régional d'Ethique de la Recherche pour la Santé Humaine du Centre of Cameroon (0120/CRERSHC/2024/), and Comité National d’Ethique pour la Recherche en Santé of Senegal (2024/0430MSAS/CNERS/SP). In accordance with ethical requirements and local data protection regulations, all data collected, including recordings, will be stored securely on access-controlled media in each country and on Université Laval’s secure platform, Valeria, which includes the REDCap (Research Electronic Data Capture) web-based software [40] REDCap provides audit trails for tracking data manipulation, and export procedure issues related to data sharing and sovereignty will be addressed through interinstitutional collaboration agreements that involve administrative, legal, and financial services from all partner institutions. These agreements will be grounded in the principles of equitable partnership, ensuring mutual recognition of data ownership, transparent decision-making processes, and fair access to research outputs across all participating countries. Informed consent will be obtained from each participant before data collection (sociodemographic questionnaire, focus groups, and interviews) begins. Participants will also be informed that they may refuse to answer any question or withdraw from the interview at any time without any negative consequences. The data collected will be anonymized to respect confidentiality and protect participants’ information. The various research documents will be coded, and only the researchers in charge of the project will have access to the list of names and codes. The information obtained as part of our study will be published in an anonymous, aggregated form so that individual responses cannot be identified and will not be passed on to third parties. To compensate for expenses incurred and constraints experienced, such as time and travel (1 hour per participant), participants will receive a lump sum according to the group to which they belong and the local context of the target countries, whether it is a woman/girl, a health care professional, or a representative of a community organization.

### Capacity Building

Our focus on capacity building throughout the project will be demonstrated by involving master’s students and other trainees from each country in developing project activities. This also includes monthly scientific discussions to share lessons learned from each country and to enhance global knowledge and practices in SDM. Additionally, we will prioritize publications and research dissemination on the topic, focusing on sharing experiences and assisting other teams working on SDM in partnerships between LMICs and high-income countries.

## Results

Funding for this project was obtained in February 2023. Interinstitutional collaboration agreements have been signed with partner institutions to formalize roles, responsibilities, and shared commitments. The steering committee has been formed in each country. The ethics boards, namely the Comité Régional d'Ethique de la Recherche pour la Santé Humaine du Centre of Cameroon, the Comitê de Ética em Pesquisa da Escola Nacional de Saúde Pública and the Comissão Nacional de Ética em Pesquisa of Brazil, and the Comité National d’Ethique pour la Recherche en Santé of Senegal gave their approval on March 13, 2024, July 15, 2024, and December 2, 2024, respectively. Data collection began in January 2025 in Cameroon. In September 2025, data collection was complete in Cameroon, 47 of the 50 participants were enrolled in Senegal, and the funds transfer was finalized in Brazil. Consequently, data collection will begin in Brazil in October 2025. All project results are expected be published in national and/or international journals by December 2026.

## Discussion

### Principal Findings

Our study aims to codevelop a digital SDM resource platform adapted to the contexts of Brazil, Cameroon, and Senegal, providing locally relevant DSTs and instructional materials to support SDM with women and girls in these 3 countries. The ENGAGEEs project is in line with the research priorities of the United Nations Research Roadmap for the COVID-19 Recovery, including helping to eliminate gender discrimination in health and social service delivery and facilitating the engagement of women and girls from LMICs in health care decisions [8]. Our research proposal combines key areas recognized as fundamental to postpandemic recovery with our expertise in SDM and knowledge mobilization to create a social innovation that improves the engagement of women and girls in LMICs in decisions about their health and well-being. This project will be enriched by the cross-cutting objective of capacity building in SDM, particularly from the perspective of increasing equitable partnerships in health research.

First, we will integrate GBA Plus into the codevelopment of a health innovation to facilitate SDM among women and girls in LMICs. This project will contribute to addressing power relations based on gender, which, intersecting with other systems of oppression such as racism and poverty, place women in an unequal position in society, thereby undermining their ability to participate in decisions about their own health [11].

Second, the codevelopment of the ENGAGEEs SDM platform will equip women and girls in 3 LMICs and health and social service professionals to share health care decisions and improve health outcomes. The project will fill a gap related to the lack of rigorous assessment of the health decision-making needs of this population of women and girls in LMICs [7]. Some studies have examined women’s autonomy in the context of reproductive health in LMICs, but few have explored women’s autonomy in other types of health care decisions [41,42]. In addition, most studies on decision aids have been developed in high-income settings [43]. The codevelopment of suitable DSTs and instructional materials will address immediate needs, while their testing and scalability assessment will provide insights for expanding their use and developing additional long-term resources [39].

Third, all our knowledge mobilization activities will be inclusive, participatory, nonhierarchical, multistakeholder, and interdisciplinary. This means that research findings will be contextualized and formulated in a way that increases their value, reliability, and relevance for policymakers and their priorities as well as for diverse groups of people. Our knowledge mobilization activities will not perpetuate or exacerbate existing inequalities and will align with a broader social change agenda. The scaling of the ENGAGEEs SDM platform will be contingent on its potential to reduce health inequities rather than exacerbate them. This will be ensured using the ISSaQ 4.0 scalability assessment tool, which considers equity a key condition for scaling.

Fourth, one of the distinguishing features of this project is the establishment of equitable partnerships for bidirectional capacity building in SDM, as provided by its governance structure. The governance is based on the principles of fair and equitable research collaboration focusing the project on the needs, priorities, and experiences of the partner countries [44]. The project activities will consider local realities, valuing different types of knowledge and evidence, being flexible and responsive to changing contexts and needs. In addition, we will integrate diverse perspectives and expertise, ensuring open and transparent communication between partners with a view to building partnerships based on trust, mutual learning, and long-term cooperation [11].

### Potential Challenges and Mitigation Strategies

We recognize that structural inequalities may lead to significant differences in the ability of women and girls to participate in the project based on their geographic location (urban vs rural), socioeconomic status, access to resources, social norms, and decision-making power. To mitigate these inequalities, we will implement several strategies. First, we will be attentive to the timing and location of data collection and project activities, understanding that women may be available at different times of the day due to caregiving responsibilities or work obligations and may face barriers such as limited access to transportation. When necessary, we will provide financial support for community workers and participants, including transportation and other participation-related expenses. Second, we will ensure accessibility by offering assistive devices and accommodation to support women with disabilities, including those with invisible or episodic conditions and mental health challenges, not only those with visible or permanent impairments. Third, we will address digital inequalities by exploring the use of offline access options for the ENGAGEEs SDM platform. Downstream, we will adopt a lightweight content design approach, thereby avoiding large images and videos and leveraging high-performance platforms (eg, Vimeo) for video storage when necessary. We will ensure that key documents, tools, or training materials are available in formats that can be downloaded for offline use. Where feasible, we will provide devices, print versions of key materials, or work through local community organizations to facilitate access for participants with limited digital literacy or internet connectivity. One facilitating factor in our participant countries is the widespread use of mobile money transaction solutions. These measures will help ensure equitable and sustained access to project resources, even in low-connectivity environments.

We are aware of the power imbalance between research teams in high-income countries and in LMICs, including differences in access to funding, language fluency, and recognition in academic spaces. We will address these power imbalances by ensuring that local research teams have full decision-making authority over activities in their context, are equitably resourced, and are recognized as co-leads in all project outputs.

Finally, in addition to the challenges of power inequalities, we acknowledge that a multiphase, multicountry project, especially in LMICs, is likely to encounter logistical challenges. We experienced this in the loss of the research lead in Rwanda, who was recruited by his government just as we were beginning the implementation of the project. Our funding application addresses the resource constraints with mitigating strategies such as providing financial support for local research leads in African countries. This is complemented by the 25% indirect costs provided by the grant and disbursed along with the transfer of funds for project implementation in each country. The team is flexible and experienced in adapting to changing circumstances. Moreover, members of the principal research team in Canada have close connections with the research networks in the target countries. Team members have already collaborated with these research teams and share their culture and languages.

### Conclusion

This project exemplifies a deep commitment to equity, diversity, and social inclusion in collaborative research. It aims to establish research partnerships that are not only equitable but also effective and sustainable, creating a positive impact on reducing social inequalities. The governance structure, based on the principles of transparency and mutual respect, ensures collective and inclusive decision-making. Local research teams and deliberative workshops will reinforce the relevance and impact of the project. By planning to identify and prioritize needs and develop suitable SDM resources, we aim to create sustainable and contextually relevant solutions. The knowledge users in this project will come from diverse groups: women and girls and their family members, health professionals, community organizers, health policymakers, researchers from LMICs, and funders. We will ensure that the results are returned to knowledge users and have a real impact in their respective organizations. These strategies have proven to be keys to success in building strong and lasting partnerships.

## Abbreviations

DST: decision-support tool
ENGAGEEs: ENGAger les femmes et les filles du Sud Global dans les décisions concernant leur santé et leur bien-être pour la mise en œuvre de la décision partaGÉE
GBA Plus: Gender-Based Analysis Plus
ISSaQ: Innovation Scalability Self-Administered Questionnaire
LMICs: low- and middle-income countries
REDCap: Research Electronic Data Capture
SDM: shared decision-making

## Data Availability

The datasets generated and analyzed during this study will be available from the corresponding author on reasonable request.
